# Changing tides: the impact of crisis on advertising

**DOI:** 10.1177/1329878X20951996

**Published:** 2021-02

**Authors:** Cameron Jenyns

**Affiliations:** Deakin University, Australia

**Keywords:** advertising, authenticity, crisis, media, messaging, strategy

## Abstract

A short essay considering the impact the Coronavirus Crisis is having on advertising. By reflecting on the industry’s response to prior economic crises and identifying key trends and approaches, this essay argues the important role authenticity offers in developing strategic advertising campaigns that challenge the orthodoxy, and make deeper, more relevant connections with consumers, resulting in enriched brand meaning and competitive advantage.

Advertising and modern economies have been conjoined complementary companions for roughly the last 170 years. As business cycles see eras of economic growth and expansion, followed by recessionary contractions – anywhere from small blips to cataclysmic collapses – advertising finds itself bolted securely onto the rollercoaster ride of the fortunes and failures of many of its largest producers. We know from previous experiences of economic crises in late modernity that marketing budgets, and particularly advertising spends, are the canaries in the shaft, leading the indicators of expenditure contraction. But for much of recent history, recessions have been getting both shorter and fewer (Tellis and Tellis, 2009). However, the economic downturn forced across swathes of the world by the COVID-19 pandemic is hardly a typical economic event.

Many commentators have been calling this a ‘once-in-a-century’ event. The negative impact to gross domestic product (GDP) has been severe for many countries fraught with the health challenge the novel coronavirus has perpetuated. Although much has been done to alleviate this, much work remains. Let us consider Australia’s current position. The Coronavirus Crisis has seen GDP per capita crumble – 0.7% (Australian Bureau of Statistics (ABS), 2020) in the first quarter in 2020. The second quarter will be much, much uglier no doubt. Some reports suggest that it could even be a 10+% contraction. The Reserve Bank of Australia’s latest Forecast Table – May 2020 – *‘Baseline’ Scenario* suggests a – 15% drop in overall consumer spending by June (Reserve Bank of Australia, 2020). This cannot bode well for advertising, as spends in Australia have reportedly plummeted by over 35% in April (Pash, 2020) alone. While overall spending was drastically down, much like the impact of COVID-19 across the world, it is unevenly spread across advertising categories. Some industries are being hammered and many others are feeling the pinch, and yet others have seen a tick upwards, such as a surge in groceries (even beyond the panic buying of toilet paper). As a reflection of the unbalanced effect COVID-19 has had on the economy, ad spends have grown year-on-year for certain categories, such as government (with its array of COVID-related initiatives and health warnings), furnishings and homewares, computers, supermarkets, beverages – alcoholic, household products and cleaners, and garden (Pash, 2020) – all favourable when lockdowns leave the vast majority of the population homebound. The categories under extreme pressure, though, have countered this growth and drawn the entire advertising output into negative territory for the period: that is a heavy weight to bear.

Although it is common to see advertising spends shift downwards coinciding with GDP contractions, not spending at all can be problematic in its own right. Following the Great Depression, Roland Vaile undertook comparative analysis at the end of the 1920s with data from advertisers who stopped advertising, others who remained on budget and those who increased their budget. Common sense might suggest the latter would be foolish, but the evidence disagreed. Increased spends saw increases in market share (Vaile, 1927). This was again tested by Alex Biel and Stephen King (2003) who found similar results during recessionary contractions in more contemporary economic climates.

But this is no ordinary recession.

This is a crisis prescribed by doctors, epidemiologists, virologists and public health experts. Credit has not frozen like the economies saw around the world during the Global Financial Crisis, nor does it have a resemblance of the early 1990s recession with domino effects of high inflation and even higher interest rates resulting in high unemployment. Furthermore, all three events – the Great Depression, the early 1990s recession and the Global Financial Crisis – saw significant stock market falls that took years to recover. But the unique aspects of this crisis have been the enormous disparity of effect with the Coronavirus Crisis and the buoyant stock markets. Planes have been grounded. Restaurants closed. Gyms doors shut. Hardware stores are open, supermarkets are overflowing and delivery service apps are running hot. These acute and unbalanced impacts raise questions about the orthodoxy of advertising expenditure during a recession. If this recession (or worse, depression) is different to those mostly inflicted by financial instruments and a crisis of confidence, will these economic oddities challenge our prior knowledge and offer new learnings for advertising strategies in the time of economic contraction? These are times where ‘creative destruction’ (Schumpeter, 1942) leads to renewal and will likely offer new insights into ways to address advertising during such times.

As William Ziff Jr. (1992) wrote in an article in the *Journal of Advertising Research* after the early 1990s recession,

. . . cyclical downturns reveal the underlying strengths and weaknesses of industries and ideas. That’s what recessions, though painful, are for – to shake out marginal activities and out-worn ideas that we’ve permitted ourselves, without noticing, during the cover of boom times. (p. RC2)

Things will change. Working from home will become more accepted, and increasing trends like online media consumption and streaming video-on-demand (SVOD) will grow faster than before, and yet other patterns are accentuated in the opposite direction, such as magazine and newspaper (hard copy) declining readership (Watson, 2020). Even radio has felt the sting of the Coronavirus Crisis (Watson, 2020), as working from home means no commute – an important contributor to listening patterns. Some of these changes in behaviour may be irreversible. Australia, for example, saw these impacts manifest early through News Corporation’s decision to cease printing 100 titles and closing 14 altogether (Kelly, 2020). Not only are these losses of employment, but also shifts in advertising opportunities with the wholesale migration to digital.

The importance of advertising during a crisis (particularly a downturn) is evidenced, but *what* we communicate matters just as much. Many advertisements need to be informational during this crisis. Consider the current advertisements of public service announcements in Australia such as ‘Stay COVID free and do the 3’, ‘Physical distancing is working’ and ‘Staying apart keeps us together’ (Department of Health, 2020a, 2020b; Victorian Government, 2020). They are functional and rational and offer clear instructions and guidance in how to personally respond to the impacts and threats of COVID-19 through informational approaches. These are similar in recommendations based on the AIDS crisis of the 1980s, another deadly transmissible disease but with vastly different communicable transmission processes and communities afflicted. However, as researchers Bush and Boller (1991) noted, ‘it is far easier to persuade individuals (over the course of an advertising campaign) to purchase a particular brand of soap than it is to persuade them to change their entire system of behaviors concerning personal hygiene’ (p. 35). Think of the straightforwardness in some of the messaging and its direct call-to-action: *Wash your hands for 30* *seconds. Keep 1.5* *m apart. Stay home*.

We also know *how* we communicate will likely change through this crisis. The literature indicates the language of rhetoric and persuasive communications changes when a crisis occurs. While many brands move to reduce their communications altogether, others change in their message approach, and particularly their tone. In the extant literature, Lee et al. (2011) look back to the Global Financial Crisis to review message strategies of financial service advertisers. The research revealed that advertisers shifted their approaches from transformational to informational. Furthermore, Lee, Taylor and Chung argued that message strategies should change to reflect the economic conditions they are required to communicate in, or, as Ziff (1992) suggests, ‘when the world changes, and our paradigm does not, the result is trouble’ (p. RC5).

Many ads have already had COVID-19-related messaging strategies reflect the context they are communicated in; these range from informational messages concerning social distancing requirements to aspirational messages like *#allinthistogether*. But are all advertisers in this together?

Many are and they are making COVID-based advertisements to build connections with their current consumers and target audiences alike. Take the Bank of America, for example – a financial institution, who, if following previous orthodoxy, should pivot from aspirational to informational. However, in the case of their recent execution ‘A Commencement for America’ (Bank of America, 2020), this is not so – narrated with a powerful story of resilience and ambition, alongside an abundance of built locations that come back to life as the lights flicker and illuminate a shutdown economy reopening after the first wave. By not following the orthodoxy of previous crises, is this an example of a shifting paradigm suitable for the Coronavirus Crisis?

To develop an understanding of what may lie ahead, let us look back at one of the more shocking crises of the recent past – 11 September 2001. The types of campaigns that followed saw some brands lean into the patriotic voice, such as General Motors and their ‘Keep America Rolling’ campaign resulted in a jump in sales that hit a new record in October 2001 (Johnson, 2020). The transformational approach was not full of puffery, nor was it disconnected from the consumer’s experience and feelings. Not only was this advertisement relevant, but it was also authentic.

Authenticity has been an emerging concept over the last 15 years in brand management. Drawn from Gary Fine’s (2003) sociological study in the context of self-taught art, which noted ‘stories affect sales’ (p. 172), brand authenticity has now become a leading concept in brand management literature. Since Fine’s work, the concept has been developed further by scholars with work that identified the role authenticity could play in strengthening brand meaning (Batey, 2002; Brown et al., 2003; Beverland, 2009; Beverland and Luxton, 2005; Gilmore and Pine, 2007; Grayson and Martinec, 2004; Gustafsson, 2005; Leigh et al., 2006) as well as defining the concept and dimensions of brand authenticity. These defined dimensions include *credibility* (Grayson and Martinec, 2004), *heritage and tradition* (Beverland, 2006; Leigh et al., 2006), *realism* (Beverland and Farrelly, 2010), *originality and uniqueness* (Gilmore and Pine, 2007; Holt, 2002), *quality* (Napoli et al., 2014) and *motivation not driven by commerciality* (Beverland, 2006; Beverland et al., 2008).

Let us return to General Motors’ ‘Keep America Rolling’ advertisement. As the United States, and world alike, watched New York come to a standstill from the terror attacks in 2001, the advertisement promoted the idea that America would not stop. It would keep going. Keep doing what it does. With the brand being ingrained in the American consumer psyche, it could be suggested that the brand authenticity dimensions *credibility* and *heritage and tradition* were projected in this advertisement. With a 0% interest offer, *motivation not driven by commerciality* could also be demonstrable.

Now to our current crisis, and keeping with the automotive sector, let us consider Isuzu UTE Australia, a brand which has seen a significant drop in sales along with the overall steep decline in total car sales in April and May, before not only a return but an actual increase in sales in June (compared to June 2019) (Federal Chamber of Automotive Industries, 2020a, 2020b). During this time, the brand continued to advertise. With this in mind, let us compare the brand’s television commercial’s (TVC) pre-, during and re-emerging from this crisis. The four advertisements are the following: ‘Go your own way with the Waymans!’ (a 90-second spot in late 2019, featuring a family’s adventures in the LCV pre-crisis); ‘Home for now. But not forever’ (a 30-second spot in mid-April as the lockdown was imposed and advising consumers of services and COVID safe work practices at dealerships); ‘We are open’ (a 30-second spot at the end of April); and, finally, ‘Adventure awaits’ (another 30-second spot as Australia broadly started to loosen restrictions in June) (Isuzu UTE Australia 2019, 2020a, 2020b, 2020c). These advertisements project authenticity in the brand’s advertising. While most are transformational, even during the lockdown, they demonstrate *credibility, realism*, and *originality and uniqueness.*

Authenticity will be important for brands during and post the Coronavirus Crisis, as stories with sincerity and genuineness will be a valued and respected brand personality dimension (Napoli et al., 2014) during the difficult times ahead. The crisis will intensify the need for brands to present authentic brand stories to make real connections to retain and grow their consumers’ commitment. While many of us in Australia and around the world remain socially distanced, making connections has never been so important, and the literature provides a solid foundation to build the relevance between advertiser and consumer. But it is not just scholars who recognise the importance of authenticity. In a recent article in the July/August edition of AdNews (2020), *The Future of Advertising*, Andy Morely, head of marketing at Uber ANZ, suggests that ‘ultimately the biggest legacy of COVID-19 will be greater authenticity from brands’, indicating a growing acceptance in industry. What is clearly seen now, and will likely unfold in a contemporary context, is that these are changing tides for advertisers. This essay highlights that although each category has its nuances and challenges that cannot be distilled into a one-size-fits-all strategy, we have argued that we do know that now is the time to be brave and commit to media spends, and equally as important to offer authentic advertising messages. Those brands that do navigate these times with such mindsets are more likely to succeed and more likely to be at a competitive advantage when once again the economic tide turns.
